# Xerophthalmia and Nyctalopia as Presenting Signs of Vitamin A Deficiency in a Patient With Rapid Intentional Weight Loss: A Case Report and Literature Review

**DOI:** 10.1002/ccr3.70896

**Published:** 2025-09-16

**Authors:** Nasser Shoeibi, Sahel Khazaei, Mehrdad Motamed Shariati

**Affiliations:** ^1^ Eye Research Center Mashhad University of Medical Sciences Mashhad Iran

**Keywords:** conjunctival xerosis, nyctalopia, vitamin A deficiency, weight loss

## Abstract

To present a patient with xerophthalmia and night blindness secondary to vitamin A deficiency following rapid weight loss. A 34‐year‐old man presented with decreased night vision, foreign body sensation, and eye dryness. He reported a rapid intentional weight loss of approximately 34 kg over the past 4 months through a restricted diet. Slit lamp examination revealed severe dry eye and a foamy appearance of the bulbar conjunctiva in both eyes. Retinal inner atrophy was evident on macular optical coherence tomography imaging. This case highlights critical considerations for clinical practice, particularly in managing patients experiencing rapid weight loss. It emphasizes the importance of thorough nutritional assessments in individuals undergoing significant weight changes, whether due to intentional dieting, bariatric surgery, or illness. Early identification of at‐risk individuals facilitates timely intervention, including dietary counseling, supplementation, and, if necessary, referral to specialists in nutrition or gastroenterology.

**Summary**

- Screening patients who experience rapid or significant weight loss for micronutrient deficiencies is crucial, as they are at high risk of developing vitamin A deficiency, which can lead to irreversible complications such as blindness.

## 1 Introduction

Rapid weight loss, whether deliberate or unintentional, can significantly impact the body's metabolism and nutritional status. Severe weight loss increases the risk of micronutrient deficiencies, including vitamin A insufficiency, particularly when combined with restricted diets or conditions that impair nutrient absorption [1]. For instance, fat malabsorption after bariatric surgery may lead to vitamin A deficiency and deficits in other fat‐soluble vitamins [2]. Additionally, individuals with persistent gastrointestinal diseases, such as Crohn's disease or celiac disease, are at a heightened risk due to compromised nutritional absorption [3].

Vitamin A is an essential micronutrient crucial for supporting and regulating the immune system, facilitating cellular communication, and maintaining optimal eye health [4]. This fat‐soluble vitamin plays a vital role in the synthesis of rhodopsin, a protein in the retina responsible for light absorption and enabling vision in low‐light conditions. It is naturally found in foods such as liver, fish oils, and leafy green vegetables. Inadequate intake or malabsorption of vitamin A can lead to a spectrum of clinical manifestations, ranging from mild symptoms like night blindness to severe conditions such as xerophthalmia, corneal ulcers, and even blindness [5].

This case report presents a patient with xerophthalmia and night blindness secondary to vitamin A deficiency, following a period of rapid weight loss. The case underscores the importance of recognizing and addressing nutritional deficiencies in individuals undergoing significant changes in body weight.

## 2 Case History and Examination

A 34‐year‐old man complained of decreased night vision, foreign body sensation, and dryness in both eyes. He reported that these symptoms began approximately 3 months ago and had progressively worsened. He mentioned a rapid intentional weight loss of about 34 kg over the past 4 months, achieved through a restricted diet.

**FIGURE 1:**
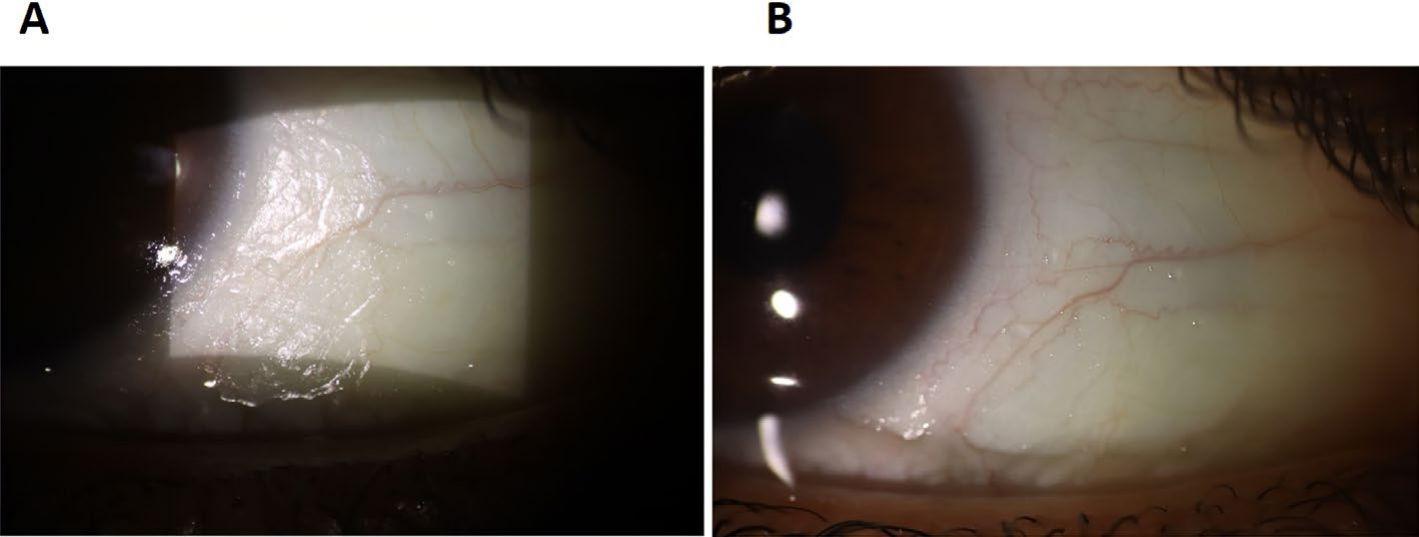
The slit photograph of both eyes (A, B) shows the Bitot spot in both bulbar conjunctivae.

Upon examination, his best‐corrected distance visual acuity was 10/10 in both eyes, and intraocular pressure was within normal limits. A slit lamp examination revealed severe dry eye symptoms, characterized by a foamy appearance of the bulbar conjunctiva and Bitot's spot in both eyes (Figure 1A,B). A Bitot's spot is a characteristic ocular manifestation of vitamin A deficiency, typically appearing as a triangular, foamy, white, or yellowish lesion on the bulbar conjunctiva, most often on the temporal side. It represents a buildup of keratinized epithelial cells and debris, resulting from squamous metaplasia of the conjunctival epithelium due to the lack of retinoic acid. Fundus examination was unremarkable.

## 3 Methods

**FIGURE 2:**
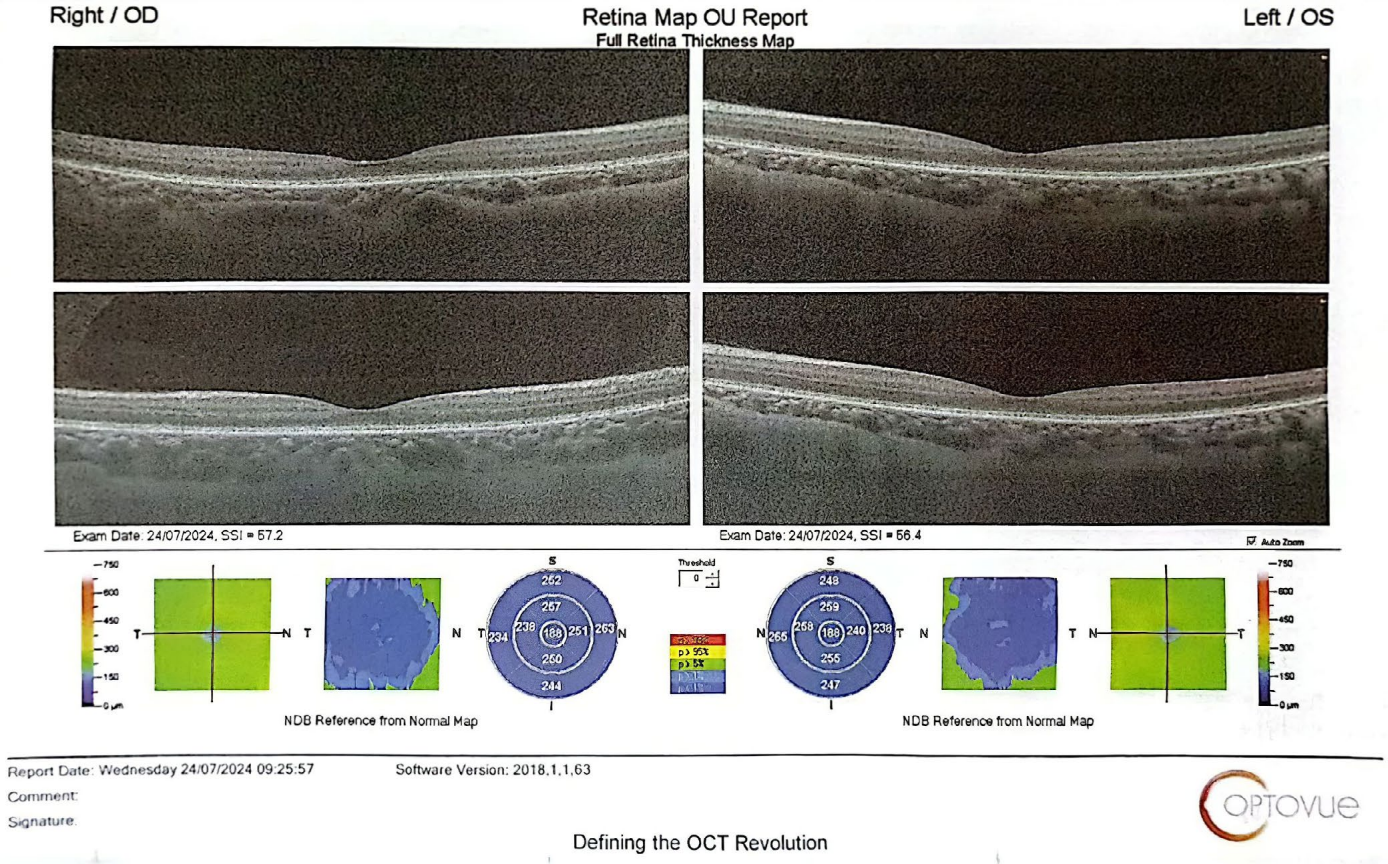
The macular OCT shows retinal atrophy in both eyes.

**FIGURE 3:**
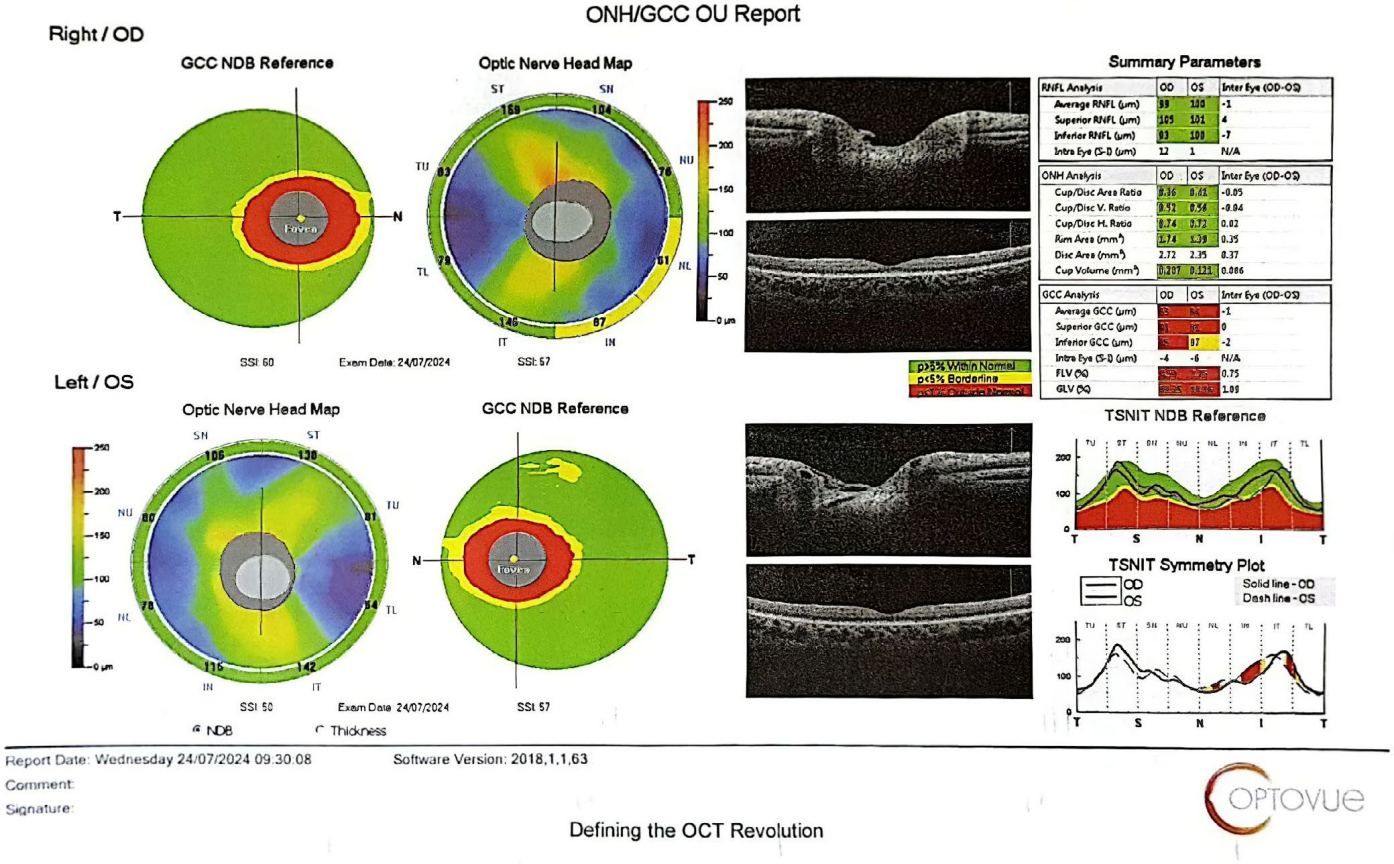
The ONH/ganglion cell complex (GCC) OCT shows perifoveal ganglion cell loss in both eyes.

We used multimodal imaging to evaluate the patient further. Spectral‐domain optical coherence tomography (SD‐OCT) (OptoVue Inc., Fremont, CA, USA, software version: 2018, 0, 0, 18) from the macula (Figure 2) and optic nerve head (ONH) (Figure 3) showed mild inner and outer retinal atrophy.

## 4 Conclusion and Results

Our findings strongly indicate that the patient was suffering from xerophthalmia and nyctalopia due to a severe deficiency in vitamin A, confirmed by serum levels measuring less than 10 μg/dL. In response to this deficiency, we initiated a treatment regimen under the guidance of a nutritionist, administering an immediate high dose of vitamin A: 100,000 IU for 3 days, followed by 50,000 IU for 2 weeks and subsequently transitioning to a maintenance dose of 5,000 IU daily. Unfortunately, the patient did not return for follow‐up examinations, limiting our ability to assess the efficacy of the treatment and monitor for potential improvements in his symptoms.

## 5 Discussion

This case represents a severe manifestation of vitamin A insufficiency, which resulted in xerophthalmia and night blindness in a patient who had lost weight rapidly. Multimodal imaging showed macular inner and outer retinal atrophy. While this illness is uncommon in wealthy nations, it remains a major global public health concern, particularly in malnutrition‐prone regions. In the presented case, the history of abrupt and dramatic weight loss was the major contributing factor to the development of vitamin A deficiency.

To further contextualize our findings, we conducted a systematic literature review using PubMed and Scopus (Supporting Information 1). Our search strategies were tailored to each database, focusing on documented cases of xerophthalmia linked to vitamin A deficiency resulting from significant weight reduction. We also reviewed the references from the included reports to ensure comprehensive coverage of the topic. This approach allowed us to gather relevant data on the prevalence, clinical manifestations, and treatment outcomes associated with vitamin A deficiency in similar patient populations. Only articles published from January 1, 2000, to February 1, 2025, were considered for inclusion in the literature review to ensure relevance to contemporary clinical practice (Table 1).

**TABLE 1:** Literature review of cases demonstrating ophthalmic manifestations of vitamin A deficiency.

Author, year	Age (year)/sex	Co‐deficiency	Underlining condition	Presenting symptom	Ophthalmic manifestation	Extraocular symptom	Treatment	Outcome
This study	34/M	None	Weight loss diet	Foreign body sensation, dryness, night blindness	Conjunctival xerosis, Bitot's spot, Retinopathy	None	100,000 IU *3 days, then 50,000 IU for 2 weeks, then 5000 IU daily	NA
Adachi, et al.,2021 [6]	7/M	Vit D, Carnitine	Malnutrition (history of autism spectrum disorder and developmental delay)	Eye rubbing, Red eye	Bilateral corneal ulcers and right corneal perforation	Phrynoderma	Oral vitamin A, D, and carnitine	Enucleation of the RE, Recovery of LE
Calikoglu, et al.,2022 [7]	34/M	NA	Biliopancreatic diversion	Vision loss	Xerophthalmia, retinopathy	NA	100,000 (IU)/d vit A, IM for 3 days, followed by ongoing IM vit A 50,000 IU/d for 2 weeks and later oral vit A 12,000 IU/d for 3 months	Full recovery
Cheshire, et al., 2017 [8]	47/F	NA	Chronic pancreatitis	Foreign body sensation, dryness, night blindness	Corneal xerosis, Bitot's spot	Cachexia	Intravenous vit A	Poor visual recovery
Chiu, et al., 2015 [9]	12/M	Folate, Iron	Malnutrition (history of autism spectrum disorder and developmental delay)	Reduced visual acuity	Corneal and conjunctival keratinization	Growth retardation	200,000 IU of vit A initially as a single dose daily for two consecutive days, followed by another dose 2 weeks later	Poor visual recovery
Crum, et al.,2018 [10]	41/F	Iron, Vit D, B12	Bariatric surgery	Dryness and diminished night vision	Corneal punctate staining, conjunctival xerosis, Bitot's spot, and retinal pigment epitheliopathy	None	8000 IU of oral vit A supplementation daily	Full recovery
Daba, et al.,2019 [11]	27/F	None	Malnutrition	Redness, tearing, photophobia	Xerophthalmia, corneal melting	Cachexia	200,000 IU vit A orally on 1st, 2nd and 7th day, Tectonic penetrating keratoplasty	Poor visual recovery
Farahbakhs, et al., 2022 [12]	2.5/M	Vit D	Cystic fibrosis malabsorption	Blurred vision, dryness	Corneal xerosis and opacity	Failure to thrive	A single oral dose of 200,000 IU vit A followed by 1500 IU per day	Partial recovery
Fernando‐Langit et al., 2008 [13]	46/F	NA	Alcohol‐induced malnutrition and cocaine dependence	Night blindness, pain	Conjunctival xerosis	Unintentional weight loss	Vit A supplementation	Full recovery
Giannaccare et al.,2020 [14]	68/F	Iron	Biliopancreatic diversion	Chronic corneal ulcer	Corneal perforation of the RE, conjunctival xerosis, and superficial punctate keratitis of the LE	NA	Gundersen conjunctival flap, Topical and IM vit A 100,000 IU, for 3 days, followed by 50,000 IU for 2 weeks	Partial recovery
Giannaccare, et al.,2020 [14]	46/F	NA	Biliopancreatic diversion	Pain and light sensitivity	Conjunctival xerosis, Bitot's spots, perforating corneal ulcer	NA	Topical and IM vit A 100,000 IU, for 3 days, followed by 50,000 IU for 2 weeks	Healing of corneal perforation, but poor visual recovery
Hsu, et al.,2015 [15]	56/F	Iron	Malnutrition	Pain, decreased vision	Conjunctival keratinization, diffuse superficial punctate keratopathy, and corneal ulcer	Skin dryness	5000 IU of vit A PO daily, amniotic membrane transplantation	Partial recovery
Lai, et al., 2014 [16]	50/M	Iron	Chronic alcohol consumption and poor dietary intake	Red eye	Bilateral corneal thinning and corneal perforation of the LE	Hepatomegaly	High‐dose oral vit A (4000 IU vitamin A/D complex)	Partial recovery
Lai, et al., 2014 [16]	48/F	Vit E	Pancreaticojejunostomy and bowel resection	Reduced vision, redness	Bilateral diffusepunctate staining of conjunctiva and cornea	None	High‐dose oral vit A, punctal plug, topical retinoic acid	Full recovery

Author, year	Age (year)/sex	Co‐deficiency	Underlining condition	Presenting symptom	Ophthalmic manifestation	Extraocular symptom	Treatment	Outcome
Lai, et al., 2014 [16]	44/M	NA	Dietary neglect	Red eye, night blindness	SPK and keratinization of conjunctiva	NA	High‐dose oral vit A, topical retinoic acid	Full recovery
Lee, et al.,2005 [17]	39/F	NA	Bariatric surgery	Red eye	Diffuse conjunctival xerosis, Bitot's spot, diffuse punctate keratitis	NA	High‐dose oral vit A (10,000 IU daily), punctal plug	Partial recovery
Madan, et al., 2017 [18]	8/M	NA	NA	Dry, itchy eye, night blindness	Bitot spots	Dry, brittle nails and skin xerosis	Oral vit A 200000 IU to be taken on 2 consecutive days, and a third dose at 14 days	Full recovery
McLaughlin, et al.,2014 [19]	41/M	NA	Poor dietary intake	Red eye, night blindness	Diffuse punctate epithelial erosions	NA	One dose of 100,000 IU of vit A IM and a regular high dose of vitamin A orally (4000 IU Vit A/D complex)	Full recovery
McLaughlin, et al.,2014 [19]	34/M	NA	Poor dietary intake	Pain, photophobia, reduced vision	Xerophthalmia	NA	Vit A supplementation	Full recovery
McLaughlin, et al.,2014 [19]	49/F	NA	Alcohol‐induced pancreatitis	Pain, blurred vision	Hurricane keratopathy	NA	Oral vit A supplementation	Partial recovery
Morjaria, et al., 2011 [20]	49/F	Vit D, zinc, iron, calcium, and magnesium	Small bowel resection	Pain, blurred vision	Bitot spots, corneal ulcer	Unintentional weight loss	Intensive multivitamins and oral vit A, along with topical gentamicin and cefuroxime eye drops	Partial recovery
Roheen, et al., 2022 [21]	2/F	NA	Malnutrition	Tearing and tonic blepharospasm	Bilateral Corneal Melting	Trichomegaly, abnormal hairs	Oral vit A 200.000 IU in divided doses for 2 days and repeated 2 weeks later	Evisceration of the RE
Sharma, et al., 2021 [22]	12/F	Vit D	Pancreatic exocrine insufficiency following Frey's surgery	Ocular irritation, photosensitivity, blurred vision	Conjunctival xerosis, diffuse superficial punctate keratitis	None	Vitamin A IM (palmitate 100,000 IU) for 2 days, followed by a third dose after 2 weeks, and then a daily dose of oral vitamin A (5000 IU per day) thereafter for 2 months, vitamin D 50000 IU once a week.	Full recovery
Anastasakis, et al.,2024 [23]	36/F	None	Bariatric sleeve gastrectomy	Reduced vision, night blindness	Reduced scotopic function	NA	Oral vitamin A supplementation for 4 weeks (10.000 IU daily)	Full recovery

Abbreviations: F, Female; IU, International unit; LE, Left eye; M, Male; NA, Note addressed; RE, Right eye; SPK, Superficial punctate keratitis; Vit, Vitamin; Wk, Week.

Vitamin A is crucial for several biological activities, including normal vision, immune function, and epithelial integrity. It is essential in rhodopsin production, a pigment molecule found in rod cells, and is necessary for seeing in low light. Vitamin A promotes epithelial cell development and mucosal surface maintenance, which is essential for infection protection [5, 24]. Ocular surface complications, such as conjunctival xerosis and Bitot's spots, are primarily attributed to a deficiency of retinoic acid, which plays a critical role in epithelial cell differentiation and maintenance. In contrast, nyctalopia and other retinal dysfunctions result from a deficiency in both 11‐cis‐retinal (a visual chromophore) and retinoic acid [25].

The body's reserves of vitamin A, mostly found in the liver, start to run low when absorption is compromised or dietary intake is inadequate. First affecting vision, the depletion compromises rhodopsin production, resulting in night blindness. The integrity of epithelial tissues is impacted when the insufficiency worsens, causing keratinization and xerosis, especially in the cornea and conjunctiva, which can develop into xerophthalmia. This disorder can lead to permanent blindness, keratomalacia, and corneal ulcers if left untreated [26].

Vitamin A deficiency is often exacerbated by conditions impairing absorption or increasing metabolic demand. For example, gastrointestinal diseases such as celiac disease, Crohn's disease, and chronic pancreatitis can significantly impair fat absorption, leading to malabsorption of fat‐soluble vitamins, including vitamin A. Similarly, conditions that cause rapid weight loss or involve severe dietary restrictions can lead to insufficient intake of essential nutrients, further increasing the risk of deficiency [27].

The relationship between rapid weight loss and vitamin A deficiency is well documented, particularly in the context of bariatric surgery and other conditions that lead to drastic reductions in nutrient intake or absorption. Rapid weight loss, whether intentional (e.g., through restrictive dieting or surgical interventions) or unintentional (e.g., due to illness), often significantly decreases overall nutrient intake. In addition, fat malabsorption may occur in cases where the weight loss is associated with gastrointestinal surgery, such as Roux‐en‐Y gastric bypass, which can impair the absorption of fat‐soluble vitamins [28].

The case presented highlights several important considerations for clinical practice, particularly in managing patients experiencing rapid weight loss. First and foremost, it emphasizes the importance of thorough nutritional assessments in individuals undergoing significant weight changes, whether due to intentional dieting, bariatric surgery, or illness. Early identification of at‐risk individuals allows for timely intervention, including dietary counseling, supplementation, and, if necessary, referral to a specialist in nutrition or gastroenterology [29]. Also, we showed that macular OCT and GCC analyses help document retinal changes secondary to vitamin A deficiency and treatment response. Berkenstock et al., in a case series in 2020, showed outer nuclear layer diffuse thinning in two patients with vitamin A deficiency, which improved with treatment [30]. Retinal structural changes in OCT are reversible in the early stages of vitamin A deficiency. However, in severe and prolonged cases, permanent atrophy of the outer nuclear layer and RPE can be seen [31].

Preoperative and postoperative nutritional assessments are critical for patients undergoing bariatric surgery, particularly those leading to malabsorption. The American Society for Metabolic and Bariatric Surgery (ASMBS) recommends routine monitoring of vitamin A levels both pre‐ and postoperatively, along with other fat‐soluble vitamins. Given the risk of deficiencies, supplementation should be considered routine, with dosages adjusted based on the type of surgery performed and the individual's nutritional status.

## Author Contributions

**Nasser Shoeibi:** conceptualization, data curation, supervision, writing – review and editing. **Sahel Khazaei:** data curation, investigation, writing – original draft, writing – review and editing. **Mehrdad Motamed Shariati:** conceptualization, data curation, visualization, writing – original draft, writing – review and editing.

## Ethics Statement

Written informed consent was obtained from the patient.

## Consent

Written informed consent was obtained from the patient to publish this case report and any accompanying images. A copy of the written consent is available for review by the Editor‐in‐Chief of this journal.

## Conflicts of Interest

The authors declare no conflicts of interest.

## Supporting information


**Appendix S1:** Supporting Information.

## Data Availability

The datasets used during the current study are available from the corresponding author upon reasonable request.
