# Supplementary material for: Xerophthalmia and Nyctalopia as Presenting Signs of Vitamin A Deficiency in a Patient With Rapid Intentional Weight Loss: A Case Report and Literature Review
Source: Clin Case Rep. 2025 Sep 16;13(9):e70896. doi: 10.1002/ccr3.70896 (PMC12441006; doi:10.1002/ccr3.70896)
Supplement: Supplementary file 1 — Appendix S1: Supporting Information. [file CCR3-13-e70896-s001.docx]

| Supplementary Material 1. Literature review through a systematic search of PubMed and Scopus | | | |
| --- | --- | --- | --- |
|  |  | **Date** | **Results** |
| Pubmed | (("xerophthalmia"[MeSH Terms] OR "xerophthalmia"[All Fields] OR "xerophthalmias"[All Fields] OR "xerosis"[All Fields]) AND ("vitamin a deficiency"[MeSH Terms] OR "vitamin a deficiency"[All Fields])) AND ((ffrft[Filter]) AND (casereports[Filter]) AND (humans[Filter]) AND (english[Filter]) AND (2000:2025[pdat])) | May 2, 2025 | 13 results, 11 relevant |
| Scopus | (ALL ("Xerophthalmia" OR "corneal xerosis" OR "keratomalacia") AND ALL ( "Vitamin A Deficiency" ) AND ALL ( "Weight Reduction" OR " Weight Loss" ) AND ABS ( " case report" OR {case- report} OR " case series" OR {case-series} ) ) AND ( LIMIT-TO ( DOCTYPE , "ar" ) ) | May 2, 2025 | 12 results, 9 relevant |
